# Supplementary material for: Micromirror-Embedded Coverslip Assembly for Bidirectional Microscopic Imaging
Source: Micromachines (Basel). 2020 Jun 10;11(6):582. doi: 10.3390/mi11060582 (PMC7345240; doi:10.3390/mi11060582)
Supplement: Supplementary file 1 [file micromachines-11-00582-s001.zip › Micromirror_SI_revised.pdf]

Supplementary Information:

Micromirror-embedded coverslip assembly for bidirectional microscopic imaging

Dongwoo Lee, Jihye Kim, Eunjoo Song, Ji-Young Jeong, Eun-chae Jeon, Pilhan Kim and Wonhee Lee*

Supplementary Figures

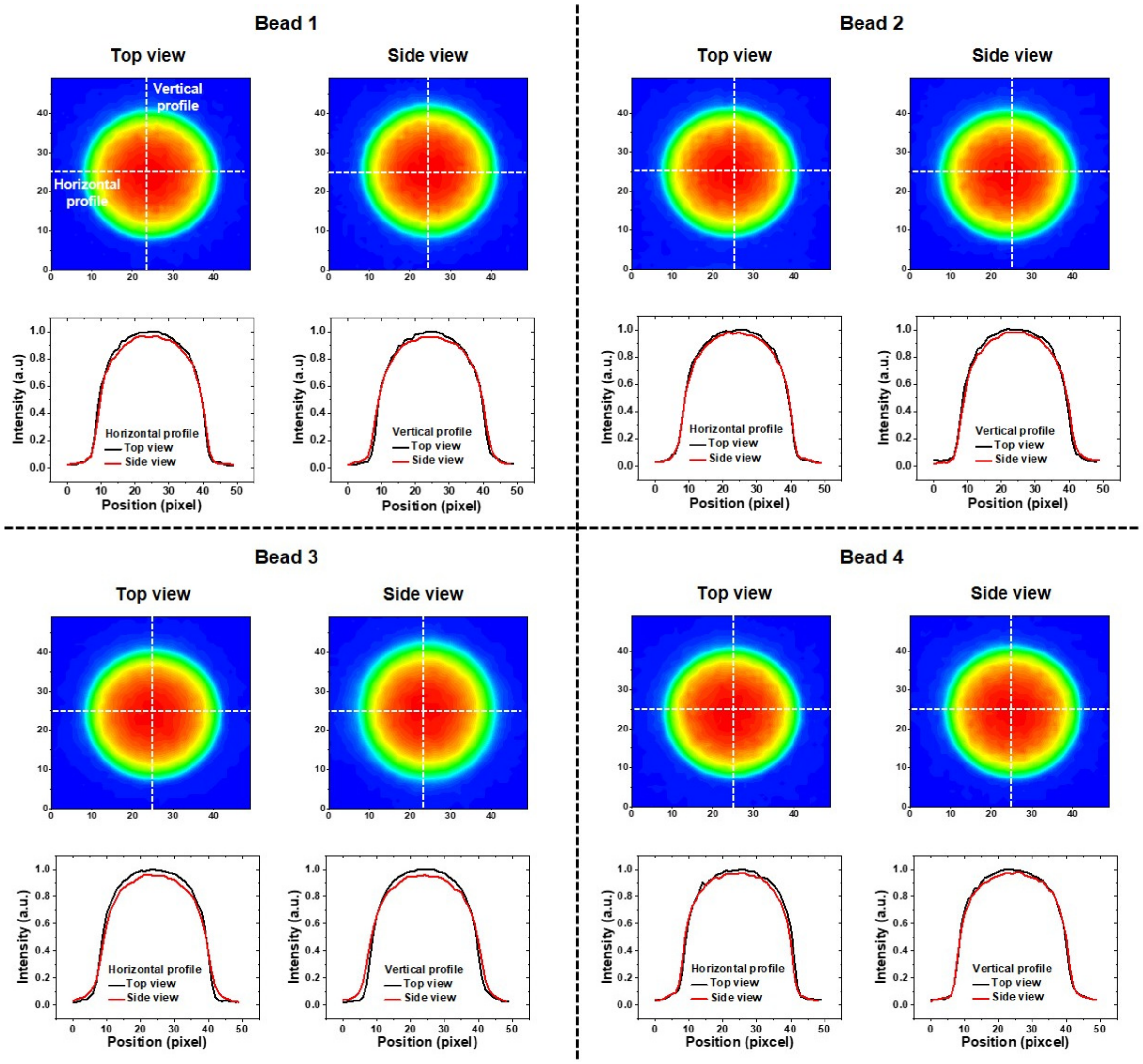

Figure S1. Intensity contours and intensity profiles in top-view and side-view of fluorescent beads.

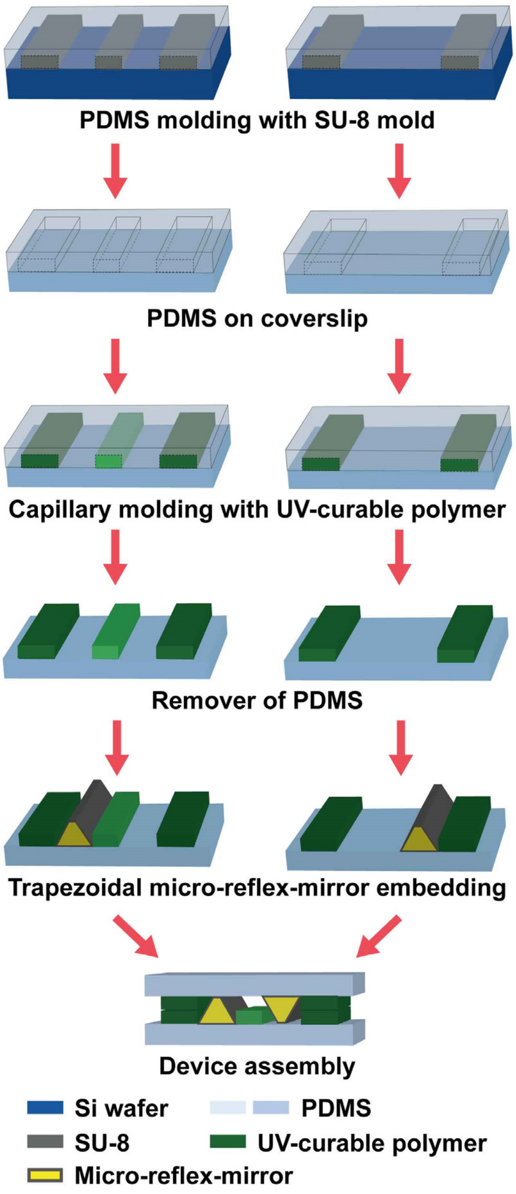

Figure S2. Fabrication steps of the micromirror-embedded coverslip with a stage for imaging of red blood cells.
